# Genital ulcers caused by sexually transmitted agents^☆^^☆☆^

**DOI:** 10.1016/j.abd.2022.01.004

**Published:** 2022-07-20

**Authors:** Mauro Cunha Ramos, Maria Rita Castilhos Nicola, Natália Tenório Cavalcante Bezerra, José Carlos Gomes Sardinha, Julia Sampaio de Souza Morais, Antônio Pedro Schettini

**Affiliations:** a Sanitary Dermatology Outpatient Clinic, Secretaria Estadual de Saúde do Rio Grande do Sul, Porto Alegre, RS, Brazil; b Fundação Alfredo da Matta, Instituição ligada à Secretaria de Estado de Saúde do Governo do Estado do Amazonas, Manaus, AM, Brazil; c Liga de DST, Faculty of Medicine, Universidade Federal Fluminense, Niterói, RJ, Brazil

**Keywords:** Chancre, Chancroid, Granuloma inguinale, Herpes simplex, Sexually transmitted diseases, Syphilis

## Abstract

Genital ulcers (GUs) represent a diagnostic challenge and can be secondary to neoplastic and inflammatory processes of different causes. Among those of infectious etiology, there are sexually transmitted infections (STIs), a very frequent reason for seeking the health service. The most common agents are herpes simplex virus and *Treponema pallidum* and, more rarely, *Haemophilus ducreyi*, *Klebsiella granulomatis* and *Chlamydia trachomatis*. A careful dermatological examination offers important diagnostic elements; however, atypical manifestations are very common. Distinctive characteristics of ulcers to look out for include their margin, edge, bottom, and base. Regional lymph node chain alterations should be evaluated regarding their number, size, mobility, consistency, inflammation, and pain on palpation. Diagnostic tests have variable sensitivity and specificity, and molecular tests are currently considered the reference exams. The rapid immunochromatographic tests represented a significant advance, as they can be performed with blood obtained from the digital pulp, offer results in up to 30 minutes, and do not require a laboratory structure. The treatment of persons affected by GU/STIs must be immediate, as it aims to prevent complications, as well as reduce transmission. It is not always considered that people with GUs/STIs have varying degrees of depression, anxiety, and self-reproach, with an impact on relationships. Establishing a bond and trusting the professional is essential for adherence to treatment and preventive measures that must be discussed individually.

## Introduction

Ulcers or ulcerations are breaks in skin continuity that reach the dermis and repair with scar formation.^1^ “Genital ulcer” is the name used by national and international authors to identify the breaks in skin continuity in the genital and perigenital region caused by sexually transmitted agents (GUs/STIs), even if at some point during their evolution they are erosions and, therefore, repair without scar formation.^2^ These lesions are a common cause for seeking medical attention and can pose a diagnostic challenge.^3^ Other causes of genital ulcers include other infectious conditions, non-infectious inflammatory processes, and neoplasias. Among the causes of infectious processes are cytomegalovirus, Epstein-Barr virus with or without signs of mononucleosis, *Mycoplasma pneumoniae*, deep mycoses, mycobacteriosis and mixed infections, such as Fournier's gangrene, an extremely severe ulcerative condition that affects the genital region.^4^ Traumatic lesions (including factitious ones), drug eruptions, and allergic or irritative contact dermatitis are also found. Fixed drug eruption is a diagnosis that is very often overlooked by physicians and difficult to understand by patients. Erythema multiforme, lichen planus, lichen sclerosus, pyoderma gangrenosum, Crohn's and Behcet's diseases may also present with ulcerated lesions.^5^ The most frequently found primary neoplasias are basal cell and squamous cell carcinomas. Melanomas, metastatic lesions, and those secondary to myeloproliferative diseases are less common but have high morbidity and mortality.^4^, ^6^ The most common agents of GUs/STIs are herpes simplex virus (HSV) and *Treponema pallidum* (TP). Much less frequent are *Haemophilus ducreyi*, *Klebsiella granulomatis*, and *Chlamydia trachomatis*. Other agents may eventually be transmitted by the sexual route, including *Entamoeba histolytica*, which can also affect other systems.^7^ There is also the potential association of different agents in the same lesion. Mixed chancre, also called Rollet's chancre, in which both *T. pallidum* and *H. ducreyi* are present, is often mentioned.^2^

The psychosocial impact of STIs is often overlooked. The affected persons almost always experience embarrassment and varying degrees of self-depreciation, depression, and anxiety. For this reason, the clinical history must be collected using an empathetic approach, in a private environment, and with the guarantee of confidentiality. The anamnesis should include collecting information about the initial characteristics of the lesion or lesions, its duration, and evolution, presence of symptoms, sexual practices, and exposures, including the identification of those potentially accountable for transmission. Previous treatments should be carefully investigated, as well as the use of other medications and drugs.^8^ Careful examination of the skin or mucosal ulcers can often reveal some distinctive characteristics. Lesion diameter and depth, characteristics of the margin, base, edge, and bottom must be observed. The affected region should be examined for scarring from previous lesions and regional lymphadenopathy. Lymph nodes should be evaluated for their number, size, mobility, firmness, inflammation, and pain at palpation.^6^

**Figure 1:**
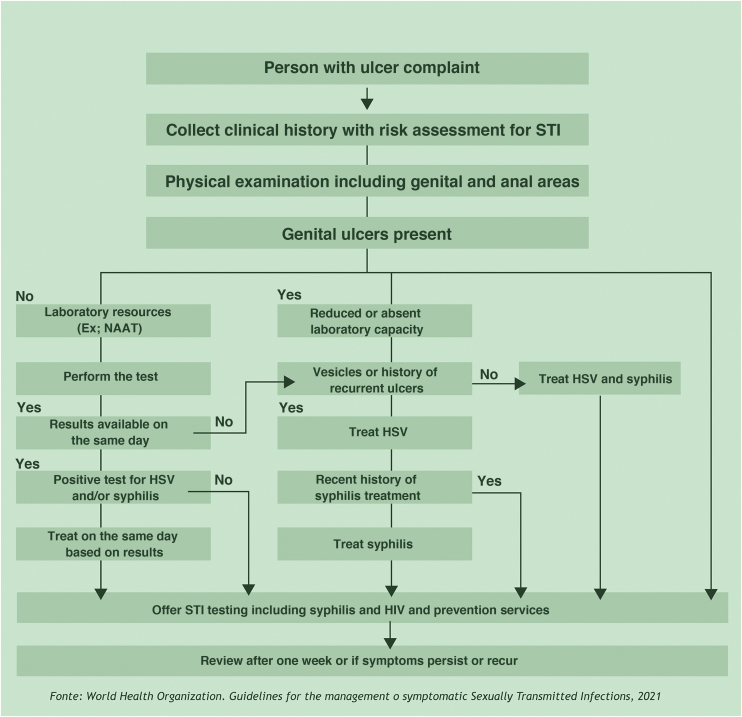
Flowchart for treating persons with genital ulcers.

GUs/STIs require treatment at the first visit to the health service.^8^ Their morphological characteristics are very important, but the diagnosis based exclusively on clinical aspects has sensitivity and specificity that are far from ideal. It is considered unacceptable to postpone treatment until the results of diagnostic tests are obtained, with the exception of rapid tests whose results can be obtained even during consultation. If an immediate diagnosis is not feasible, the presumptive diagnosis is justified by the flowchart-based approach. The prompt establishment of treatment reduces complications and transmission. Fig. 1 shows the flowchart proposed by the World Health Organization.^9^ The known spontaneous regression of lesions, difficulty in accessing health services, an impediment to being absent from work, and psychosocial conditions make patients fail to return to the medical service. Care services for people with STIs should facilitate the conditions for return consultations, either by establishing a relationship of trust or through an adequate organization.

GUs/STIs represent a significant behavioral marker of risk for HIV infection.^10^, ^11^ Additionally, the breaching of the skin-mucosal barrier and the greater influx of HIV infection target cells, including CD4+ lymphocytes, increase viral transmission. The presence of treponema and its constituents, for instance, induces the expression of the CCR5 protein, an HIV receptor in macrophages and T-cells.^12^ Moreover, syphilis negatively interferes with the course of HIV infection, just as HIV infection leads to greater severity in syphilis manifestations.^13^

## Hard chancre

It is caused by *Treponema pallidum* subspecies pallidum, being the primary manifestation of syphilis, which is a chronic, multisystemic disease with a variable course that can lead to severe sequelae or death. The bacterium ranges in length from five to 15 micrometers and is approximately two micrometers wide. It shows rotational movement on its longitudinal axis and undulation that propagates from one extremity to the other.^14^ It is not Gram-stained but can be identified by the Giemsa, Vago (methyl violet), and silver stainings.^15^ It is maintained by inoculation into rabbits and is often referred to as non-culturable. However, its viability and molecular characteristics were maintained for more than 6 months in culture media under strict homeostasis conditions.^16^ It consists of an outer, cytoplasmic membrane and endoflagella connected to nanomotors at the poles, which are responsible for its motility. Its invasiveness, which occurs through microabrasions on the skin or intact mucous membranes, is associated with the adhesion capacity and the proteolytic activity of the TP0751 protein, and its evasiveness to the immune system is due to the low protein content of its outer membrane.^13^ The infection quickly becomes systemic and often invades the central nervous system, but this fact does not lead to an inevitable progression to neurosyphilis.^14^ *T. pallidum* does not produce exotoxins or endotoxins, and the manifestations are a consequence of the host immune response, resulting from a delayed type IV hypersensitivity reaction.^17^ This reaction produces a dense perilesional inflammatory infiltrate, consisting of lymphocytes, macrophages, and plasma cells.^14^

### Epidemiology

The disease is transmitted primarily through sexual intercourse of any kind, including oral sexual activity and intimate skin contact with the exudate from open lesions. It can also be transmitted from the infected mother to the fetus at any time during pregnancy or delivery. A third and rarer form of transmission is the parenteral one^15^; however, the screening for infections in blood and organ donors eliminates this possibility.^18^ The World Health Organization estimates a global annual incidence of six million cases of acquired syphilis.^19^ High-income countries have shown an increase in syphilis cases in the last two decades. In the US, for instance, the incidence of primary and secondary syphilis increased by 71% between 2014 and 2018, with 90% of these cases occurring in men and 82% in Men who have **S**ex with Men (MSM). Ethnic minorities are also more affected^20^ and, in women, it is six times more frequent in injectable drug users or partners of users.^11^ Low- and middle-income countries are much more severely affected and, although there is a disproportionately high incidence in MSM, it occurs in young individuals of both sexes, regardless of sexual orientation. In Brazil, the incidence rates of acquired syphilis have tripled, increasing from 19.7 per 100,000 inhabitants to 72.8 per 100,000 in 2019. Approximately half of the reported cases of acquired syphilis occurred in women, a fact that results in a severe incidence of 8.2 cases of congenital syphilis/1,000 live births in Brazil.^21^

### Clinical manifestations

**Figure 2:**
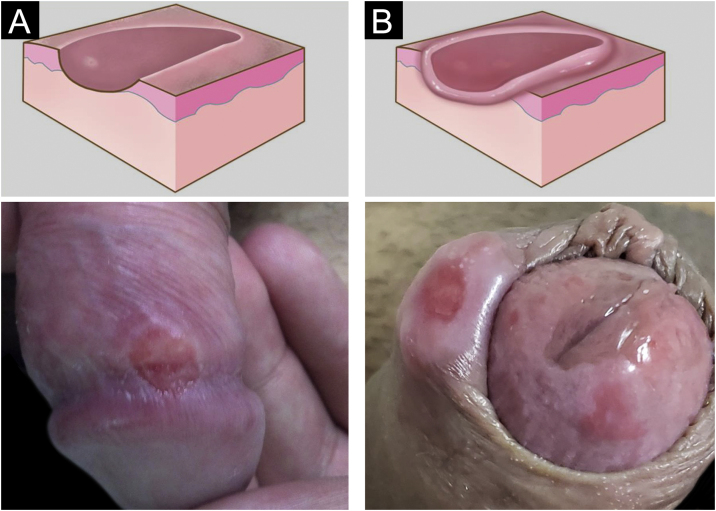
(A), Eroded recent hard chancre, rounded with smooth sloping edge; (B), Hard chancre with evident infiltration. Graphic illustration adapted from wikicommons.org (public domain).

Hard chancre occurs at the point of the treponema inoculation. The incubation period can range from nine to 90 days, most commonly two to three weeks. The classic lesion begins as a small macule or papule that erodes and typically becomes a single, well-defined, round, or oval ulcer.^22^ Its bottom is usually clear, red and bright at times (Fig. 2a).^23^ It has a smooth sloping edge, accompanied or not by an elevated edge. The base is infiltrated with a cardboard-like or cartilaginous consistency (Fig. 2b). It is usually referred to as painless, but this is a subjective and highly variable symptom.

**Figure 3:**
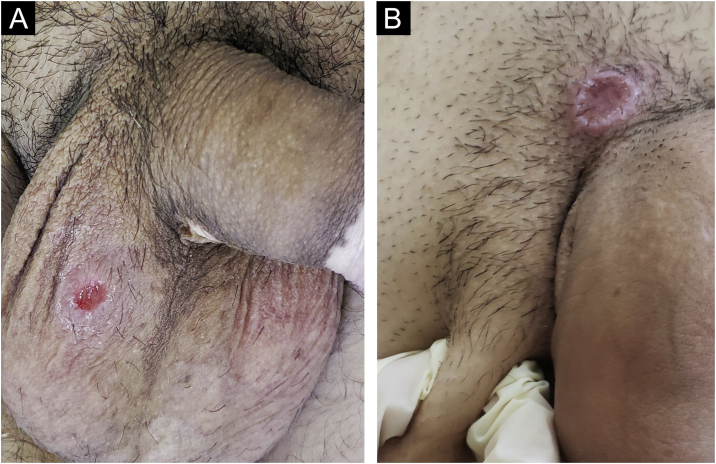
(A), Hard chancre on the scrotal skin; observe the clear bottom. The patient also has vitiligo. (B), Lesions on the pubis, near the base of the penis, with a cupuliform appearance, reflecting the infiltration of the base. Palpable regional lymphadenopathy can be observed.

**Figure 4:**
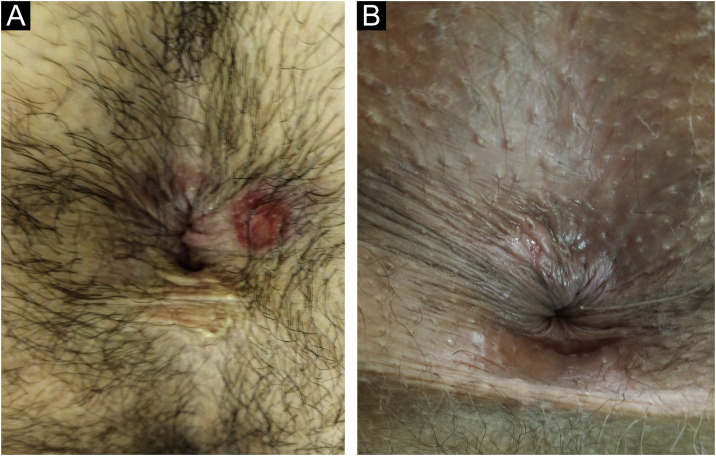
(A), Perianal hard chancre with typical configuration. The color of this lesion is often compared to that of raw ham. (B), When close to the anus, it may have an irregular shape, following the structure of the anal folds.

**Figure 5:**
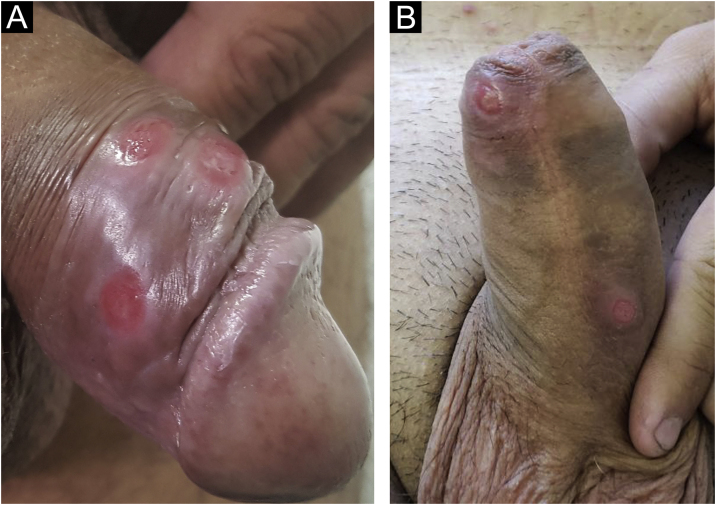
(A and B), Multiple hard chancres may occur in up to 25% of cases but are more commonly found in HIV-infected patients.

**Figure 6:**
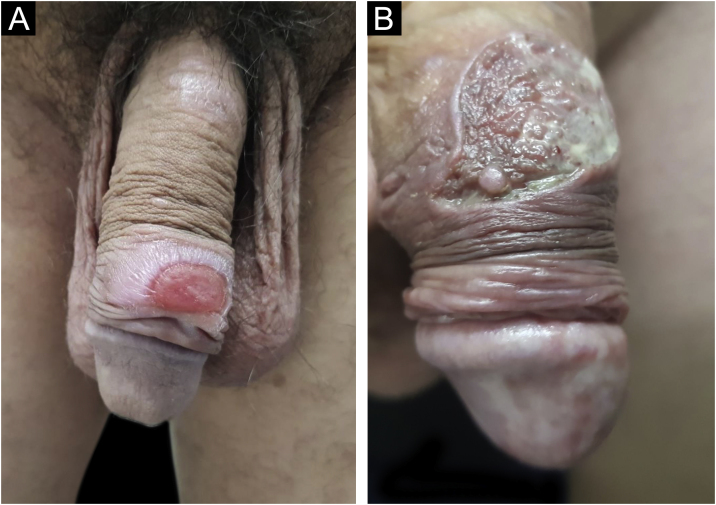
(A), When the lesions are larger than two centimeters in diameter, they are called giant chancres. (B), When destructive, they are called phagedenic chancres, often associated with superinfection by associated bacteria.

**Figure 7:**
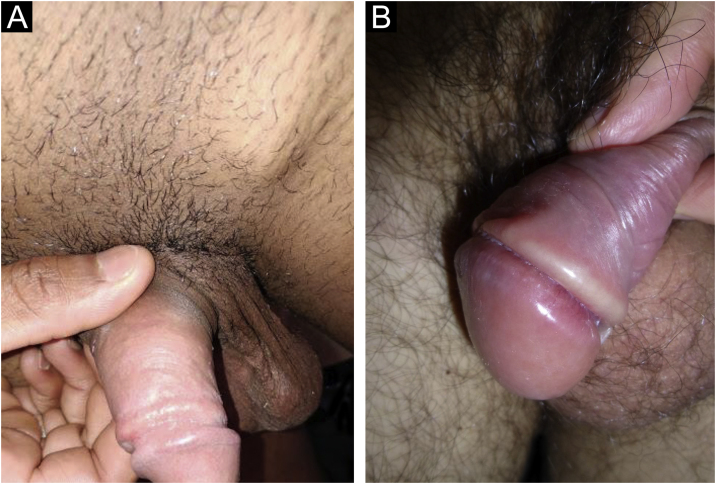
(A), Regional lymphadenopathy accompanies cases of hard chancre. They comprise multiple lymph nodes with a larger one: the “mayor node”. (B), Cord-like lesions may mimic Mondor's disease or transient lymphangiectasia of the penis.

In men, it is often found on the glans, coronal sulcus, frenulum and the inner leaflet of the foreskin. It is less frequent in the skin of the penis, scrotum (Fig. 3a) and pubis (Fig. 3b). In women, it is found in the labia majora and minora, in the posterior commissure of the labia minora, in the posterior commissure, in the perineum, and in the perianal region. In the perianal region, it can maintain the typical configuration (Fig. 4a), and the lesion often has a color that is compared to that of raw ham. Also in the perianal region, when closer to the anus, it may show an irregular edge and an almost linear shape, following the structure of the anal folds (Fig. 4b). Because it is often painless and hidden in cavities or little visible places, it often goes unnoticed. More rarely, it may occur at other sites on the skin such as the nipples and fingers.^22^ Multiple lesions can occur in up to 25% of the cases (Figs. 5a and 5b) and are more commonly found in patients with HIV infection. More rarely, it has larger dimensions, sometimes called giant chancre (Fig. 6a), with more destructive characteristics, sometimes called a phagedenic chancre (Fig. 6b).^24^ These occur mainly in patients with different forms of immunosuppression, including older age, malnutrition, and alcoholism. Without treatment, the lesion spontaneously disappears, within three to six weeks, almost always without scar formation. Regional lymphadenopathy is virtually universal and usually has a “rubbery” firmness (Fig. 7a). It consists of multiple lymph nodes, to which the name “*la pléiade de Ricord*” has been attributed.^2^ One of these lymph nodes is larger and has been referred to as the “mayor ganglion”.^24^ The classic description of the chancre is constant in the literature, but the occurrence of uncharacteristic forms is frequent. In a series of 64 patients with primary syphilis, only 42.7% had classic ulcers.^25^ Non-ulcerated primary lesions occur much more rarely, including cord-like lesions (Fig. 7a), which may mimic Mondor's disease or transient penile lymphangiectasia (Fig. 7b).^26^, ^27^

### Diagnosis

The definitive diagnosis is based on the direct demonstration of *T. pallidum* in the lesion exudate. Dark-field microscopy examination allows the diagnosis before the production of antibodies in sufficient quantity for serological detection. It should be carried out immediately after collection, while treponemas maintain their mobility; the results depend a lot on the observer's experience. In a study carried out in Valencia, Spain, its use allowed the definitive diagnosis by dark-field microscopy in 429 patients with primary syphilis. Of these, 48% had negative serological tests at the time.^25^ It cannot be used in the investigation of oral cavity lesions due to the presence of *Treponema denticola*, a constituent of the local microbiota and morphologically indistinguishable from *T. pallidum*. Smear microscopy using Fontana-Tribondeau, Burri, Giemsa or Levaditi stains is rarely used. Direct immunofluorescence, despite its excellent sensitivity and specificity, is seldom used, due to the difficulty in obtaining supplies.^22^

DNA amplification tests, including polymerase chain reaction (PCR) are also considered definitive, as they detect specific sequences of *T. pallidum*. In a recent meta-analysis, the specificity of PCR in primary syphilis samples was estimated at 80%‒96% and the sensitivity at 78.4% (95% CI 68.2–86.0). The genes used as targets in these reactions include tpp47, bmp, polA, and 23S rRNA, which had similar performances.^28^

Treponemal and non-treponemal serological tests are effectively used in practice for the presumptive diagnosis and therapeutic decision-making. A list of the most common tests is shown in Table 1. Treponemal tests detect antibodies against antigenic components of *T. pallidum* itself. They become positive earlier than non-treponemal ones and have better sensitivity and specificity. These tests, however, cannot differentiate active from a previous infection.^5^ The clinical and epidemiological history is very important for therapeutic decision-making. The rapid tests by immunochromatography, also treponemal ones, represent a great advance in the diagnostic arsenal, as their performance is superior to that of tests carried out in the laboratory and provide results in up to 30 minutes.

**Table 1:** Treponemal and non-treponemal tests.

Non-treponemal	Treponemal
Venereal Disease Research Laboratory (VDRL)^a^	Rapid tests (immunochromatographic tests; RT)^a^,*
Rapid plasma reagin (RPR)^a^, *	*Fluorescent Treponemal Antibody Absorption Test* (FTA-Abs)^a^
Toluidine Red Unheated Serum Test (TRUST)	*T. pallidum Haemagglutination test* (TPHA)
	*Micro-Haemagglutination Assay for T. pallidum* (MHA-TP)^a^
Unheated Serum Reagin (USR)	*Enzyme Linked Immunonosorbent Assay* (ELISA)^b^
	*T. pallidum* passive particle agglutination test (TPPA)^b^
	Chemiluminescence Immunoassay for *Treponema pallidum* (CLIA)
	Chemiluminescent Microparticle Immuno Assay (CMIA)

a Tests most frequently used in Brazil.

b No laboratory equipment required.

* Automated tests, with increasing use in laboratories with a large volume of samples.

Nontreponemal tests, in turn, detect anticardiolipin antibodies and can be titrated. The most often used are the VDRL (Veneral Disease Research Laboratory) and the RPR (Rapid Plasm Reagin). The latter is easier to perform and is equally effective. In addition to being used for diagnosis, they are also used for post-treatment monitoring; however, they should not be used interchangeably in the follow-up of the same patient, as they do not have comparable titers. Once one of the two tests is used, it must be used until the follow-up is completed. They become reactive in about two weeks after the infection and their reactivity decreases in later forms of the disease. Although very rare, false-positive reactions can occur in diseases such as leprosy, chronic hepatitis, malaria, autoimmune diseases and, exceptionally, during pregnancy.^8^ False-negative results may occur when large amounts of antibodies are present (prozone phenomenon).^22^

### Treatment

The drug treatment of syphilis is shown in Table 2.

**Table 2:** Drug treatment of genital ulcers caused by sexually transmitted agents.

STI	Clinical condition	First choice	Alternative
Herpes simplex	First episode	Acyclovir 200 mg 2 tablets orally every 8 hours for 7 to 10 days or 200 mg 1 tablet orally every 4 hours, while awake for 7 to 10 days. Start as early as possible.	Valacyclovir 1 g orally, every 12 hours for 7–10 days^c^
	Recurrence	Acyclovir 200 mg, 2 tablets orally, every 8 hours, for 5 days or 4 tablets orally, every 12 hours for 5 days. Preferably start in the prodromal period.	Valacyclovir 500 mg orally every 12 hours for 3 days^c^
	Suppression (≥6 episodes/year or high psychosocial impact)	Acyclovir 200 mg, 2 tablets orally, every 12 hours, for up to 6 months. The use can be extended for up to two years with sporadic assessment of kidney and liver functions	Valacyclovir 1 g orally once daily for up to 6 months or Valacyclovir 500 mg orally once daily. The use can be extended for up to 2 years with sporadic assessment of kidney and liver functions
	Pregnancy	Treat first episode in any trimester. If there was a primary infection or if the recurrences are frequent during pregnancy, suppressive therapy can be performed as of the 36^th^ week	Insufficient data for use during pregnancy
Syphilis	Hard chancre	Benzathine penicillin 2.4 million IU, IM, in a single dose (1.2 million IU in each gluteus)	Doxycycline 100 mg^a^^b^, 1 tablet orally, every 12 h for 15 days
	Syphilis with duration ≥1 year or with unknown duration	Benzathine penicillin 2.4 million IU, IM, every 7 days for 3 weeks (1.2 million IU in each gluteus). Total dose of 7.2 million IU	Alternative: doxycycline 100 mg, every 12 h orally, for 30 days
Chancroid		Azithromycin 500 mg, 2 tablets, orally in a single dose	Ceftriaxone 250 mg, IM, single dose
Lymphogranuloma venereum		Doxycycline 100 mg^a^^b^, 1 tablet orally, every 12 h for 21 days	Azithromycin 500 mg, 2 tablets every 7 days for 21 days (preferential for pregnant women)
Donovanosis		Doxycycline 100 mg^a^^b^, 1 tablet orally, every 12 hours or Sulfamethoxazole with trimethoprim (400/80 mg), 2 tablets orally, every 12 hours. Both at least for three weeks	Azithromycin 500 mg, 2 tablets orally, every 7 days for at least three weeks or until the lesions heal.

IU, International Units; IM, Intramuscular.

a Contraindicated in pregnancy. The only treatment considered adequate for syphilis in pregnancy is penicillin.

b Source: *Ministério de Saúde do Brasil. Protocolo Clínico e Diretrizes Terapêuticas Infecções Sexualmente Transmissíveis* (2015, update 2020). Brasilia; 2020.

c CDC. Sexually Transmitted Infections Treatment Guidelines. Atlanta. 2021.

## Genital herpes

Two types of herpes viruses are recognized as causing GUs, herpes simplex type I (HSV-I) and herpes simplex type II (HSV-II). They are members of the *Herpesviridae* family and the simplex virus genus and share 40% of their genomic structure. They consist of a large double-stranded DNA molecule, an electron-dense nucleus that contains the viral DNA, and an icosapentahedral capsid wrapped in a layer of proteins, which is called the integument and is covered by the envelope.^29^

Infection occurs through contact of the virus with the skin surface, especially with the oral and genital mucosa. The virus infiltrates the skin through micro-fissures, replicates in the epidermis, and penetrates the cutaneous endings of the sensory nerves. It then travels along neuronal axons and reaches the nucleus of neurons in the sensory ganglia of the spinal cord. In these, it can remain latent for months or years. During reactivation periods, it descends along the same sensory nerves to the skin surface, replicating in epidermal cells, producing inflammation and lesions.^30^, ^31^

### Epidemiology

It is estimated that approximately one in six people aged between 14 and 49 years have a genital herpes infection in the United States.^29^ Fifty to 80% of GUs/STIs are caused by HSV. It was believed that HSV-I had a predilection for the orofacial region, and type two for the genital region. It has, however, been demonstrated that both regions can be affected by either type, especially in high school students, young heterosexual women, and men who have sex with men. This change in epidemiology can be attributed to the increase in unprotected oral sexual practices. Recurrences are known to be more frequent in HSV-2 infection.^30^, ^31^

### Clinical manifestations

**Figure 8:**
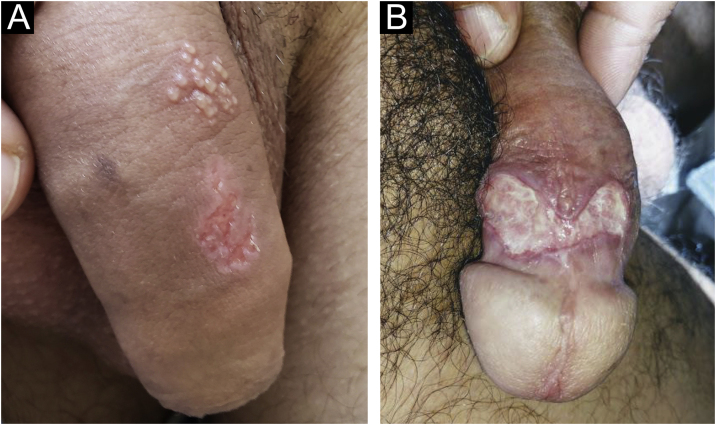
(A), Herpes simplex in subentrant outbreaks: grouped vesicles on an erythematous base and a superficial ulcer undergoing healing with a polycyclic edge. (B), Deeper ulcer with a fibrinoid bottom and undermined edge.

Most HSV infections are asymptomatic and when they show cyclical manifestations, they do so in the same anatomical region, alternating with periods of latency of variable duration.^32^ These episodes are commonly associated with periods of physical or emotional stress, fever, medication use, solar radiation, menstruation, or even sexual activity. Some patients have a more severe initial condition, usually called primary infection. The resulting lesions are much more painful, numerous, and affect much larger areas. In women, dysuria or urinary retention and low back pain, simulating an upper urinary tract infection, are not uncommon. Not infrequently, fever and general malaise occur. At the reactivations, many patients report the occurrence of prodromes characterized by pruritus, pain, or paresthesia. These symptoms are followed by the appearance of vesicles with citrine or yellowish content, grouped “in clusters” on an erythematous base. The rupture of the vesicles leads to the appearance of erosions or ulcers with a well-defined edge, which coalesce and show a polycyclic configuration, reflecting the coalescence of small round lesions, secondary to the vesicles (Fig. 8a). Deeper and more persistent ulcers may occur in immunosuppressed patients (Fig. 8b). The lesions are painful, especially on manipulation, and are often accompanied by equally painful regional lymphadenopathy. When in humid areas, the vesicles are hardly evident, as they rupture early due to epithelial maceration. They occur in any location on the genital or perigenital regions. On the face, they occur on the periorificial areas, more commonly on the lip vermilion. They are less common on other areas of the skin and may be called *herpes gladiatorum* because of their more frequent occurrence in those who practice contact sports.^32^

### Diagnosis

Cytodiagnosis using the Tzanck method can be very useful, although its sensitivity can be variable, depending on the type of collection. The craped material, obtained from the exposed floor of a previously intact vesicle or recent erosions, should be placed on a slide and stained through the Giemsa, Leishman, or Hematoxylin-eosin methods. It is considered positive when giant and multinucleated epithelial cells are identified. The detection of viral antigens through direct immunofluorescence can be used. Viral culture is reserved for research laboratories and DNA amplification tests, particularly the polymerase chain reaction, are currently considered the reference exams. Serological testing is generally not very useful, since a positive result may indicate a previous infection and not necessarily active HSV disease. Histopathology is usually reserved for cases of difficult diagnosis, atypical or chronic ones. When performed, it initially reveals the presence of keratinocytes with ground-glass nuclei, and cytoplasmic swelling and pallor. This is followed by “ballooning” degeneration of keratinocytes, resulting in intraepidermal vesicles containing multinucleated giant cells with molding of the nuclei.^33^

### Treatment

Drug treatment of genital ulcers caused by HSV can be found in Table 2.

## Lymphogranuloma venereum

It is caused by *Chlamydia trachomatis* of the serotypes L1, L2 and L3. Chlamydia is an immobile, intracellular growing bacterium. The microorganism has the capacity to penetrate the skin and mucous membranes. Once in the cytoplasm, it undergoes binary fission until its perinuclear microcolonies promote cell lysis and spread through the lymphatic vessels. It can cause localized or systemic disease and affect different organs, including those of the genitourinary tract, most notably causing salpingitis, as well as the gastrointestinal tract, lungs, and joints.^34^

### Epidemiology

Lymphogranuloma venereum (LGV) was described by Wallace in 1883 who, believing it was a climate-related disease, called it “tropical bubo”. It has also been called the “fourth disease”, Durand-Nicolas-Favre disease, and was commonly known as “mule”. It is an uncommon STI, whose degree of infectivity and potential disease reservoirs are not well known. Its transmission has been largely attributed to asymptomatic carriers.^35^ It is thought to be responsible for two to 10% of genital ulcers in Africa and Southeast Asia.^36^ It has become more frequent in European countries since 2003 among Men who have Sex with Men (MSM). The L2b variant with high clonal affinity has been identified in most of these cases^37^ and it is hypothesized that it was imported from the USA in the end of the last century. It is estimated that 25% of anorectal infections are asymptomatic in this population^35^ and most commonly manifest as proctitis, with the ulcerative disease being rarer.^38^ Transmission among MSM population can be partially explained by anogenital contact, as the ratio of genital LGV infection *versus* anorectal infection is 1:15.^39^ Transmission through sexual activity between men and women is a rare event in these continents.^40^

### Clinical manifestations

The presentation depends on the inoculation site. The incubation period varies from one to four weeks and the disease has three stages. In the primary stage, it starts with a small papule or pustule that, when eroded progresses to a small ulcer. The lesion is usually painless and disappears within a week, which is why it goes unnoticed; very rarely it is a cause for seeking medical care and, even more rarely it is identified during clinical examination.^36^ When inside the rectum, the urethra, or the vagina, mucopurulent discharge may occur.^40^

The secondary stage starts between two and six weeks after the end of the primary stage and manifests as regional lymphadenopathy. In the case of inoculation in the external genitalia, it affects the inguinofemoral region, with the formation of a typical lateral and extremely painful bubo. The inflammation progresses to fluctuant swelling and drainage in approximately one-third of patients. Some patients develop the groove sign or Greenblatt's sign, in which the inguinal ligament is identified as a depression that separates the inguinal and femoral lymph nodes. In developing countries, this is the typical presentation of LGV, particularly in men.^41^ The main manifestation of LGV in epidemic MSM in Europe is proctitis, characterized by evident symptoms of anorectal pain, bloody purulent discharge, tenesmus, constipation and, at times, “flat stools”. This clinical picture is sometimes difficult to differentiate from that of rectal cancer and, clinically and histopathologically, from Crohn's disease.^42^

In women, the rectum, upper vagina, cervix or urethra may be affected, regions where the lymphatic drainage occurs to the deep iliac or perirectal lymph nodes, resulting in lower back and lower abdominal pain, which may be accompanied by low-grade fever, chills, malaise, myalgia, and arthralgia.^36^

The tertiary stage also called “anogenital-rectal syndrome,” is more frequent in women and starts with the development of proctocolitis, followed by abscesses, fistulas, strictures, and stenosis of the rectum.^40^ Progressive lymphangitis leads to chronic edema and sclerosing fibrosis, resulting in strictures and fistulas in the affected region, culminating in the appearance of elephantiasis, which when located in the female external genitalia, is called esthiomene.^43^ Rare reactive complications include reactive arthritis, cardiac involvement, aseptic meningitis, and inflammatory eye disease.^41^ Rare septic complications include arthritis, pneumonitis, and (peri)hepatitis.^41^

### Diagnosis

Nucleic acid amplification tests (NAAT) specific for *C. trachomatis* can be performed with the material collected from the exudate of the primary ulcerated lesion or from bubo aspirates. Detection techniques, such as culture and immunofluorescence can also be used. Serological tests do not differentiate between the serotypes and histopathological examination of a lymph node sample does not show specific findings.^44^

### Treatment

Drug treatment can be found in Table 2. Buboes with fluctuation and liquefaction can be aspirated by puncture through healthy skin using a syringe with a large-caliber needle. Incision and surgical drainage are not recommended due to the possibility of permanent fistula formation. In cases of residual fibrotic lesions with the destruction of the architecture, surgical repair, including reconstruction of the genital tract, should be considered.^8^

## Chancroid

Ulcus molle or chancroid is a sexually-transmitted ulcerative disease that primarily affects the genital and perigenital regions.^1^ It is caused by *H. ducreyi*, which is a bacillus with rounded extremities, measuring approximately 1.2 to 1.5 µm in length and 0.5 µm in width. It is a facultative anaerobic, gram-negative, immobile, non-spore-forming bacterium. On Gram stain, it shows typical streptobacillary chaining. It is fastidious in culture, requires the presence of hemin (factor X) for its growth and all its strains reduce nitrate to nitrite when tested. The streptobacillary configuration in two parallel chains is compared to “railway tracks” or to clusters in “fish schools”.^4^ The sensitivity and specificity of these configurations, however, are not sufficient for species determination.^5^

### Epidemiology

Chancroid is disappearing even in countries that maintained epidemic levels, with the exception of Malawi and northern India.^45^ *H. ducreyi* is usually transmitted through sexual activity, and more recently it has been identified as the cause of extragenital skin ulcers in children in Oceania and the South Pacific.^46^ There are, however, two elements that hinder the understanding of global epidemiology: lesions caused by herpes simplex are frequently and erroneously assumed to be caused by *H. ducreyi*, and most studies do not use sufficiently sensitive and specific methods for the diagnosis.^47^ A recent systematic review analyzed 49 studies on chancroid, of which 35 were published between 1980 and 1999, and 14 between 2000 and 2014. In the first studied period, the proportion of genital ulcers caused by *H. ducreyi* ranged from 0% in Thailand and China to 68.9% in South Africa. In the second later period, it ranged from 0 in Namibia, Mozambique, Zambia, Brazil, and Australia to 15% in Malawi. The authors summarize the data, indicating a sustained decline over the past few decades.^47^ Chancroid has become rare in high-income countries. In the USA, for instance, seven cases were reported in 2017. The substantial decrease in prevalence occurred after the introduction of syndromic management for the treatment of GUD by the WHO, and major social changes after the year 2000. In Brazil, the only prospective study using PCR to identify the etiological agents of genital ulcers in 434 patients was performed in Manaus in 2013 and found no cases of chancroid.^48^

### Clinical manifestations

**Figure 9:**
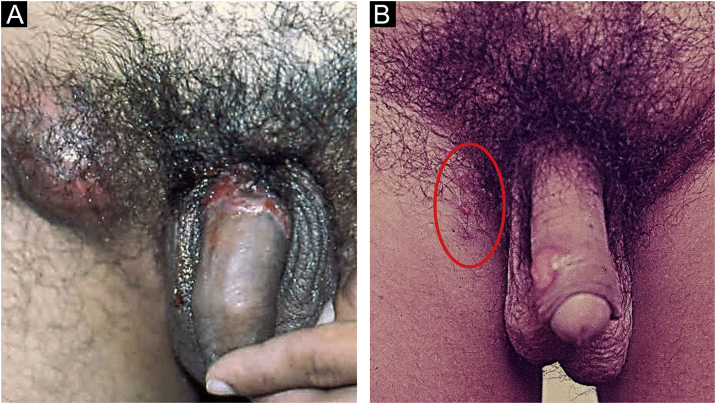
(A), Chancroid: non-infiltrating, serpiginous and undermined ulcers. “Dirty” bottom and painful, usually unilateral, inflammatory inguinal lymphadenopathy. (B), Ulcers by reinoculation. Clinical pictures belong to Prof. Sinésio Talhari private collection.

The infection can be asymptomatic in both sexes, but the lesions caused by *H. ducreyi* are usually extremely painful, preventing sexual activity. Chancroid incubation period is short. Three to seven days after the infection, painful, erythematous papules develop, most often on the inner leaflet of the foreskin or frenulum in men and on the vulva, cervix, or perianal area in women. These can cause vaginal or anal discharge or bleeding. They quickly develop into pustules that, when they rupture, originate ulcers whose edges are described as irregular, serpiginous and undermined (Fig. 9a). The bottom of the ulceration is often friable and covered by pyonecrotic exudate. The base does not show infiltration, except in the case of secondary infection. Self-inoculation results in the so-called “*cerco* ulcers” (Fig. 9b), as well as ulcers in the fold areas, described as “in book pages”. As a complication, gangrene in extensive areas of the skin or even penile gangrene may occur. Inguinal lymphadenitis, which develops in approximately half of the cases, is usually unilateral and very painful (see also Fig. 9a). Not infrequently, they develop into lesions with fluctuation that can drain spontaneously, usually through a single orifice.^8^

### Diagnosis

The sensitivity and specificity of the identification of *H. ducreyi* through optical microscopy are not sufficient for a definitive diagnosis or exclusion of chancroid. Culture in special media is a very specific test; however, as it is an extremely fastidious bacterium, the sensitivity is low, about 80%. It is useful for the identification of bacterial resistance in epidemiological studies. PCR, although not always available, is the most sensitive and specific diagnostic method. Exudate collection should be performed using a cotton swab to vigorously rub the lesion. Multiplex DNA amplification kits that simultaneously detect other pathogens, such as *Treponema pallidum* and herpes simplex can also be used.^49^

### Treatment

Drug treatment can be found in Table 2. Treatment seems to be less effective in uncircumcised men and patients coinfected with the human immunodeficiency virus (HIV). Sexual partners should be treated for chancroid, regardless of symptoms, if they have had sexual contact with the patient in the previous ten days.^50^ Treated patients usually show symptom improvement within three days and objectively within seven days after therapy. Clinical resolution of the lymphadenopathy is slower and may require needle aspiration. Some authors admit the possibility of performing an incision and drainage.^45^, ^51^

## Donovanosis

### Bacteriology/Etiopathogenesis

Donovan described intracellular inclusions in 1905, which were later called “Donovan's bodies”. The disease is also called granuloma inguinale and is caused by *Klebsiella granulomatis*, an intracellular, Gram-negative, facultative anaerobic bacterium.^52^, ^53^ It was later reclassified based on its phylogenetic similarity to other *Klebsiella* species.^54^ Its pathogenesis remains largely unknown, due to its fastidious characteristics and difficult culture.^55^

### Epidemiology

Donovanosis shows low infectivity. There are cases in which no definite history of sexual contact is identified. However, sexual transmission seems to be the main mode of transmission: it is more frequent in the genital and perigenital region in sexually active young adults and is often associated with other sexually-transmitted diseases.^56^ Poor hygiene and low socioeconomic status are risk factors and incidence data are very scarce.^57^ It has been described as endemic in tropical countries such as India, South Africa, Indonesia, Papua New Guinea, the Caribbean, Australia, the southern United States, Argentina, and Brazil, but the reliability of these data is highly questionable.^58^

### Clinical manifestations

The incubation period has not been fully established, but most sources mention a period of up to 40 days. Although genital and perigenital lesions are much more common, accounting for 90% of cases, lesions in the cephalic segment, face, trunk and armpits, joints and bones are rarely described. Internal organs may also be affected, particularly in immunocompromised patients.^58^

**Figure 10:**
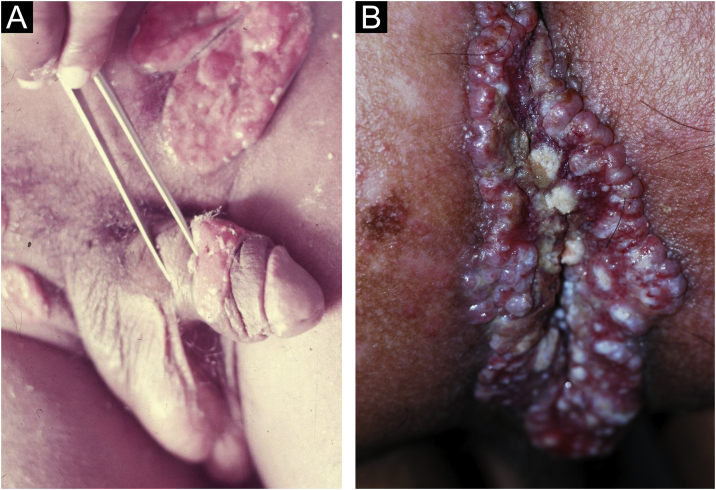
(A), Donovanosis: Vegetative ulcer with a bright red bottom (raised and/or everted edge). (B), Linear growth along, which may reach large dimensions, along the skinfolds. Clinical pictures belong to Prof. Sinésio Talhari private collection.

The manifestations of genital and perigenital lesions, are very variable, and Lobo-Jardim in 1987^59^ proposed the following classification: 1) Ulcerous lesions, 2) Ulcerous-vegetative (most frequent form), 3) Vegetative and 4) Elephantiasis. They are usually painless and do not have satellite lymphadenopathy. However, pseudo-buboes (subcutaneous granulomatous infiltration) may occur. In men, they are preferentially located on the coronal sulcus, balano-preputial region, anus, and perianal region. In women, it most commonly affects the labia minora and the vaginal fourchette. The involvement of the cervix and upper genital tract may mimic neoplastic processes.^56^ It initially presents as a papule or subcutaneous nodule, which develops into a vegetative ulcer with an elevated or slightly everted edge, with a fleshy (bright red) bottom (Fig. 10a). Linear growth along the skin folds is common, reaching large dimensions (Fig. 10b) and, as the lesion progresses at one extremity, it regresses leaving an atrophic scar at the other. Chronic disease can cause tissue destruction and genital elephantiasis.^5^

### Diagnosis

The identification of Donovan bodies, through direct microscopy, in smears collected directly from the lesion remains the most effective method for confirming the diagnosis. For cytological examination, the material should be collected from areas of active granulation without signs of secondary infection. An imprint smear can also be made by pressing the biopsy specimen obtained for histopathological examination against the slide.

Giemsa, Leishman, or Wright stains are used to better highlight the presence of the bacteria.^60^ A biopsy can be very helpful. Examination through hematoxylin-eosin staining demonstrates epidermis with marked and irregular acanthosis, with a pseudoepitheliomatous appearance that must be differentiated from epithelial carcinomas. The dermis is entirely occupied by a dense inflammatory infiltrate, consisting of histiocytes, lymphocytes, plasma cells and, less commonly, eosinophils and neutrophils. When present, neutrophils form microabscesses. Donovan bodies can be seen within vacuolated histiocytes, especially with silver stains such as the Warthin-Starry staining method.^53^

### Treatment

Drug treatment is shown in Table 2.

## Final considerations

GUs/STIs are a frequent cause for seeking medical care, both in specialized and in primary care units. Despite being crucial, the clinical aspects do not always allow a definitive diagnosis. Laboratory investigation can qualify epidemiological surveillance measures, allowing the identification of isolated or associated pathogens and their susceptibility to antibiotics, allowing the rational use of drugs. DNA amplification techniques can be employed for the screening of infections in asymptomatic individuals, especially in populations at higher risk. It is important for the medical professional to be acquainted with the diagnostic tests characteristics, especially with their sensitivities and specificities, and positive and negative predictive values. Many tests rely on sophisticated laboratory facilities, rarely available in resource-poor settings, and the delay in obtaining the results precludes prompt treatment. Therefore, the tests that are really useful for the management of symptomatic individuals are the rapid tests, which allow their use during the consultation. Clinical guidelines with treatment flowcharts can be very useful, but they require validation according to local epidemiologic and systematic updating.

All partners of individuals diagnosed with a STI should be evaluated and, if indicated, adequately treated. In many situations, especially in the case of syphilis, chancroid, and lymphogranuloma venereum, epidemiological treatment should be strongly considered and discussed with these patient partners. The notification criteria can be found at http://www.aids.gov.br/pt-br/profissionais-de-saude/ist/pcdt-ist and the individual notification form of the Notifiable Diseases Information System (Sinan) can be found at http://portalsinan.saude.gov.br/images/documentos/Agravos/NINDIV/Notificacao_Individual_v5.pdf.

Counseling for risk reduction is part of the care for persons with sexually transmitted infections. There is no standardized risk reduction program for everyone. It is important to provide the information that the association between different STIs is frequent and that they facilitate the transmission of HIV. For these reasons, it is important to systematically recommend testing for syphilis, HIV, hepatitis B, and C and, considering regional epidemiology, for HTLV. Among the measures that should be considered are: limiting the number of sexual partners, and correct and consistent use of male or female condoms during sexual intercourse. This use must be demonstrated and promoted.^8^ Condom use reduces but does not eliminate the risk of transmission of sexually transmitted agents that cause GUs. Intimate contact with areas not covered by a condom and oral sexual activity can result in transmission. The three most commonly implicated pathogens (HSV-1, HSV-2, and *T. pallidum*) can be efficiently transmitted by oral sex.^51^ This information is generally unknown to patients. Male circumcision is considered protective and associated with reduced risk for STIs.^61^ The authors believe this procedure should be discussed, especially with patients who repeatedly visit medical services services due to STIs. HIV pre-exposure prophylaxis (PREP) is well established for persons at high risk or partners of HIV-infected persons. The biannual evaluation with screening for syphilis and other STIs is part of the PREP protocol. Recent studies have evaluated the efficacy of using doxycycline in pre-or post-exposure,^62^ but they have a short follow-up period and the establishment of selective pressure and the development of resistance to the utilized drug as limitations.

## Financial support

None declared.

## Authors' contribution

Maria Rita Nicola: Statistical analysis; approval of the final version of the manuscript; design and planning of the study; drafting and editing of the manuscript; collection, analysis, and interpretation of data; effective participation in research orientation; intellectual participation in the propaedeutic and/or therapeutic conduct of the studied cases; critical review of the literature; critical review of the manuscript.

Mauro Cunha Ramos: Statistical analysis; approval of the final version of the manuscript; design and planning of the study; drafting and editing of the manuscript; collection, analysis, and interpretation of data; effective participation in research orientation; intellectual participation in the propaedeutic and/or therapeutic conduct of the studied cases; critical review of the literature; critical review of the manuscript.

Natália Tenoro Cavalcante Bezerra: Statistical analysis; approval of the final version of the manuscript; design and planning of the study; drafting and editing of the manuscript; collection, analysis, and interpretation of data; effective participation in research orientation; intellectual participation in the propaedeutic and/or therapeutic conduct of the studied cases; critical review of the literature; critical review of the manuscript.

José Carlos Gomes Sardinha: Statistical analysis; approval of the final version of the manuscript; design and planning of the study; drafting and editing of the manuscript; collection, analysis, and interpretation of data; effective participation in research orientation; intellectual participation in the propaedeutic and/or therapeutic conduct of the studied cases; critical review of the literature; critical review of the manuscript.

Júlia Sampaio Souza Morais: Statistical analysis; approval of the final version of the manuscript; design and planning of the study; drafting and editing of the manuscript; collection, analysis, and interpretation of data; effective participation in research orientation; intellectual participation in the propaedeutic and/or therapeutic conduct of the studied cases; critical review of the literature; critical review of the manuscript.

Antônio Pedro Schettini: Statistical analysis; approval of the final version of the manuscript; design and planning of the study; drafting and editing of the manuscript; collection, analysis, and interpretation of data; effective participation in research orientation; intellectual participation in the propaedeutic and/or therapeutic conduct of the studied cases; critical review of the literature; critical review of the manuscript.

## Conflicts of interest

None declared.
